# Differences in immune-related toxicity between PD-1 and PD-L1 inhibitors: a retrospective cohort study in patients with advanced cancer

**DOI:** 10.1007/s00262-024-03869-1

**Published:** 2024-11-07

**Authors:** Cecilia Olsson Ladjevardi, Marcus Skribek, Anthoula Koliadi, Viktoria Rydén, Ali Inan El-Naggar, Evangelos Digkas, Antonios Valachis, Gustav J. Ullenhag

**Affiliations:** 1 Department of Immunology, Genetics, and Pathology, Uppsala University, Uppsala, Sweden; 2 Department of Oncology, Uppsala University Hospital, 751 85 Uppsala, Sweden; 3 Thoracic Oncology Center, Theme Cancer, Karolinska University Hospital, 171 76 Stockholm, Sweden; 4 Department of Oncology-Pathology, Karolinska Institutet, 171 77 Stockholm, Sweden; 5 Department of Oncology, Faculty of Medicine and Health, Örebro University, Örebro, Sweden; 6 Department of Oncology, Mälarsjukhuset, 633 49 Eskilstuna, Sweden

**Keywords:** PD-1 inhibitors, PD-L1 inhibitors, Immune-related adverse events, Advanced cancer, Cohort study

## Abstract

Immunotherapy with PD-1 or PD-L1 inhibitors has become an essential treatment strategy for a growing number of malignancies. These treatments have a risk for immune-related adverse events (IRAEs). Pooled analyses based on clinical trials show a favorable toxicity profile for treatment with PD-L1 compared to PD-1 inhibitors. This study aimed to investigate differences in IRAEs between patients with advanced solid malignances treated with PD-L1 and PD-1 inhibitors in a real-world setting. We conducted a retrospective cohort study at four Swedish Regions. Patients (n = 605) treated for advanced cancer with a PD-L1 or PD-1 inhibitor in monotherapy between June 2016 and August 2022 were included. Non-small cell lung cancer (NSCLC) was the most common malignant disease (n = 251; 41.5%), followed by malignant melanoma (n = 173; 28.6%), renal cell carcinoma (n = 71; 11.7%) and urothelial carcinoma (n = 35; 5.8%). Among patients receiving PD-L1 inhibitors, NSCLC (94.4%) was the predominant malignancy, whereas for those treated with PD-1 inhibitor, malignant melanoma constituted the most prevalent malignancy (34.5%). Discontinuation of treatment due to IRAEs overall and IRAEs grade ≥ 2 were significantly less common in patients treated with PD-L1 compared to PD-1 inhibitors [Odds Ratio (OR): 0.38 (95% Confidence Interval (CI) 0.16–0.88) and OR: 0.63 (95% CI 0.35–0.98) respectively]. Any grade IRAE, IRAE grade ≥ 3 and multiple IRAEs were numerically more frequent in patients treated with PD-1 inhibitors.In conclusion, our study of patients with advanced solid malignancies in a real-world setting supports the results from clinical trials demonstrating a favorable toxicity profile for PD-L1 inhibitors versus PD-1 inhibitors.

**Supplementary Information:**

The online version contains supplementary material available at 10.1007/s00262-024-03869-1.

## Introduction

During the last decade, immunotherapy with checkpoint inhibitors (CPIs) has become an important treatment option for a growing number of malignancies. Programmed cell death protein-1 (PD-1) is expressed on the surface of activated T cells and serves as an essential immunological checkpoint through downregulation of immune response and prevention of autoimmunity in healthy tissue, thus functioning as a brake for immune response [1, 2]. PD-1 receptor protein binds to two ligands, programmed death ligand-1 (PD-L1) and programmed death ligand-2 (PD-L2). PD-L1 is extensively present on various hematopoietic cells (T cells, B cells, macrophages, dendritic cells) as well as non-hematopoietic cells (epithelial, stromal, and endothelial). PD-L2 is mainly found on hematopoietic cells (Th2 cells, B cells, dendritic cells, macrophages), but also on certain epithelial cells, especially in the lung [2]. PD-L1 is commonly overexpressed on tumor cells where it binds to PD-1 receptors present on T-cells causing escape of antigen specific T-cell immune response [1].

The two main groups of CPIs are monoclonal antibodies directed to PD-1 or PD-L1. Anti-PD-L1 antibodies block the binding of PD-L1 to PD-1 and B7-1/CD80, while anti-PD-1 antibodies block the binding of PD-1 to PD-L1 and PD-L2 [2]. The first PD-1 inhibitors, pembrolizumab and nivolumab, were approved by the Food and Drug Administration (FDA) in 2014 [3, 4]. Until now, FDA has approved nine monoclonal antibodies targeting PD-1 (cemiplimab, toripalimab, nivolumab, pembrolizumab, tislelizumab and dostarlimab) or PD-L1 (atezolizumab, avelumab and durvalumab) for treatment of various malignancies in different therapeutic settings [5].

Monotherapy with a PD-1 or PD-L1 inhibitor is generally well tolerated compared to chemotherapy [6, 7]. However, these treatments commonly cause side-effects called immune related adverse events (IRAEs) as a result of activation of the immune system against healthy tissue. Most IRAEs are mild and reversible when treated accurately, but some IRAEs are long-term persisting and may even be fatal [8, 9]. Due to the differences in mechanism of action between antibodies targeting PD-1 and PD-L1, it is possible that these drugs differ in treatment efficacy as well as in frequency, pattern, and intensity of IRAEs.

No randomized head-to-head study comparing the effects of PD-1 and PD-L1 inhibitors has been reported. In terms of potential differences in IRAEs, a few meta-analyses based on results from clinical trials have been conducted comparing IRAEs from treatment with PD-1 versus PD-L1 inhibitors, demonstrating increased frequency of IRAEs with PD-1 inhibitors [10–13]. As the current evidence is solely based on data from patients treated in clinical trials, the generalizability of these results in real-world populations can be questioned. Moreover, conflicting results exist on whether there are differences in IRAE frequencies and patterns between PD-1 and PD-L1 inhibitors [14–16].

The present study aimed to investigate differences in IRAEs due to treatment with PD-1 versus PD-L1 inhibitors among patients with advanced solid malignancies treated in a real-world setting.

## Patients and methods

### Study design and setting

The study is a multicenter retrospective cohort study. Patients from four regions in Sweden (Södermanland County, Uppsala County, Örebro County, Stockholm County) who were treated with PD-1 or PD-L1 inhibitors against advanced cancer were included. Patients from the Stockholm County cohort received treatment between June 2016 and August 2022, while patients from the other cohorts were treated from January 2017 to December 2021. Only patients treated with PD-1 or PD-L1 antibodies in monotherapy were included**.** Patients with concomitant chemotherapy, targeted therapy, or other immunotherapies, as well as patients treated in a curative setting, were excluded. All treatments were administered with standard doses and schedules according to international guidelines specific to the tumor type and based on clinical judgement by the treating physician.

The ESMO Guidance for Reporting Oncology real-world evidence (GROW) was followed [17].

### Data collection

Data were extracted from electronic medical records (EMR) by researchers (clinical oncologists) and entered into a database with pre-specified variables of interest. The following data were collected and analyzed: age at diagnosis, gender, comorbidities expressed as Charlson Comorbidity Index, type of cancer, primary treatment at diagnosis, age at diagnosis of advanced cancer, metastatic sites, CPI initiation date, performance status (PS; WHO classification) at CPI initiation, number of previous lines of treatment, best treatment response on CPI, date of disease progression, IRAEs (date, type, grade, outcome), date of death and cause of death. The clinical stages were defined according to the 8th edition of the TNM classification [18].

### Outcomes and definitions

The National Cancer Institute Common Toxicity Criteria for Adverse Events (CTCAE) version 5:0 grading system was used to categorize IRAEs [19]. When the grade was not specified in the EMRs, the grade was approximated based on the description of adverse events in EMRs and, when present, laboratory findings.

Multiple IRAEs were defined as IRAEs affecting more than one organ system either simultaneously or sequentially.

### Statistical methods

Patient-, tumor-, and treatment-related characteristics were presented through descriptive statistics using numbers and percentages for categorical variables and median and range for continuous variables. For bivariate comparisons, chi-square (for comparisons between categorical variables) or Mann–Whitney test (for comparisons between categorical and continuous variables) were utilized.

To investigate whether type of CPI was independently associated with frequency of IRAEs (in different clinically relevant definitions of intensity) or discontinuation due to IRAEs, multivariable logistic regression models were applied. Covariates in the models were pre-defined based on current literature and included age, gender, performance status, cancer type, treatment line, and duration of CPI treatment. For these analyses, a complete case approach was applied. For each variable, Odds Ratio (OR) and its corresponding 95% Confidence Interval (CI) is presented.

All analyses were performed using SPSS (IBM, SPSS Statistics, v.28.0).

## Results

### Characteristics of the study cohort

The baseline characteristics of the study cohort are summarized in Table 1. A total of 605 patients, with a median age of 67 years (range: 20–87) were enrolled. The sex distribution was 345 (57%) males and 260 (43%) females. Non-small cell lung cancer was the most common underlying malignant disease (251 patients; 41.5%), followed by malignant melanoma (173 patients; 28.6%), renal cell carcinoma (71 patients; 11.7%), urothelial carcinoma (35 patients; 5.8%), head and neck squamous cell carcinoma (HNSCC), (23 patients; 3.8%) and other malignancies (52 patients; 8.6%). Most patients were treated with PD-1 inhibitors (498 patients; 82.3%) and the rest were treated with PD-L1 inhibitors (107 patients; 17.7%).

**Table 1:** Characteristics of study cohort

Variable	N (%)
Age, median (range), in years	67 (20—87)
Gender Male Female	345 (57.0) 260 (43.0)
Type of cancer NSCLC Melanoma Renal cell carcinoma Urothelial carcinoma HNSCC Other	251 (41.5) 173 (28.6) 71 (11.7) 35 (5.8) 23 (3.8) 52 (8.6)
de novo metastatic disease	297 (49.1)
Visceral metastases	415 (68.6)
Central nervous system metastases	49 (8.1)
Performance status according to ECOG 0 1 ≥ 2 Missing	199 (32.9) 291 (48.1) 113 (18.7) 2
Type of checkpoint inhibitor PD-1 inhibitor PD-L1 inhibitor	498 (82.3) 107 (17.7)
Line of treatment for checkpoint inhibitors 1st 2nd 3rd or later	237 (39.2) 253 (41.8) 115 (19.0)

*Abbreviations*: NSCLC, Non-Small Cell Lung Cancer; HNSCC, Head and Neck Squamous Cell Carcinoma; ECOG, Eastern Cooperative Oncology Group; PD-1, Programmed Cell Death Protein 1; PD-L1, Programmed Death-Ligand 1

### Characteristics of patients treated with PD-1 compared to PD-L1 inhibitors

Comparisons on baseline characteristics between patients treated with PD-1 and those treated with PD-L1 inhibitors are listed in Table 2. The proportion of males treated with PD-1 inhibitors was higher than females (59.4% vs. 40.6%, *p*-value = 0.010) whereas females dominated in the group treated with PD-L1 inhibitors (54.2% vs. 45.8%, *p*-value = 0.010). The most common underlying malignancy among patients treated with PD-L1 inhibitors was NSCLC (94.4%), whereas the most prevalent malignancy for patients treated with PD-1 inhibitors was melanoma (34.5%), followed by, NSCLC (30.1%) and renal cell carcinoma (14.3%). A significantly greater proportion of patients treated with PD-L1 inhibitors had de novo metastatic disease (67.3% vs. 45.2% *p*-value < 0.001) as well as visceral metastases (80.4% vs. 66.1% *p* = 0.004). There was also a significant difference in WHO performance status (PS) between the group treated with PD-1 compared to PD-L1 inhibitors (*p*-value = 0.010). A higher percentage of patients treated with PD-1 inhibitors had WHO PS 0 (35.5% vs. 21.2%) whereas more patients treated with PD-L1 inhibitors had WHO PS 1 (60.6% vs. 45.8%). In addition, there was a significant difference in the line of treatment for PD-1 versus PD-L1 inhibitors (*p*-value < 0.010). PD-1 inhibitors were predominantly favored as first line treatments (45.2% vs. 11.2%), while PD-L1 inhibitors were more commonly used in second (57.9% vs. 38.4%) or subsequent lines of therapy (30.8% vs. 16.5%). Furthermore, the median treatment duration was significantly longer for the cohort treated with PD-1 inhibitors compared to patients treated with PD-L1 inhibitors (median 3 months vs. 2 months, *p*-value = 0.037).

**Table 2:** Comparison on baseline characteristics between patients treated with PD-1 and those treated with PD-L1 inhibitors

Variable	PD-1 inhibitors N = 498 n (%)	PD-L1 inhibitors N = 107 n (%)	*p*-value
Age, median (range), in years	67 (20—87)	70 (26—84)	0.096
Gender Male Female	296 (59.4) 202 (40.6)	49 (45.8) 58 (54.2)	0.010
Type of cancer NSCLC Melanoma Renal cell carcinoma Urothelial carcinoma HNSCC Other	150 (30.1) 172 (34.5) 71 (14.3) 35 (7.0) 23 (4.6) 47 (9.4)	101 (94.4) 1 (0.9) 0 (0.0) 0 (0.0) 0 (0.0) 5 (4.7)	< 0.001
de novo metastatic disease	225 (45.2)	72 (67.3)	< 0.001
Visceral metastases	329 (66.1)	86 (80.4)	0.004
Central nervous system metastases	38 (7.6)	11 (10.3)	0.362
Performance status according to ECOG 0 1 ≥ 2	176 (35.5) 227 (45.8) 93 (18.8)	22 (21.2) 63 (60.6) 19 (18.3)	0.010
Line of treatment for checkpoint inhibitors 1st 2nd 3rd or later	225 (45.2) 191 (38.4) 82 (16.5)	12 (11.2) 62 (57.9) 33 (30.8)	< 0.001
Duration of treatment, median (range), in months	3 (1—33)	2 (1—40)	0.037

*Abbreviations*: PD-1, Programmed Cell Death Protein 1; PD-L1, Programmed Death-Ligand 1; NSCLC, Non-Small Cell Lung Cancer; HNSCC, Head and Neck Squamous Cell Carcinoma; ECOG, Eastern Cooperative Oncology Group

### Occurrence of IRAEs with PD-1 compared to PD-L1 inhibitors

The frequency of IRAEs and their outcomes in both the cohorts of this study are shown in Table 3. IRAE grade ≥ 2 and treatment discontinuation due to toxicity were significantly more common in the cohort treated with PD-1 inhibitors compared to those treated with PD-L1 inhibitors (35.7% vs. 22.4%, *p*-value = 0.008 and 17.3% vs. 7.5%, *p*-value = 0.011 respectively). Any grade IRAE, IRAE grade ≥ 3 and multiple IRAEs were numerically more frequently observed among patients treated with PD-1 inhibitors although without statistical significance. The median time to IRAE onset was 2 months (range: 0–36 months) for PD-1 and 1 month (range: 0–26 months) for PD-L1 treated patients, respectively (*p*-value = 0.405).

**Table 3:** Frequency of immune-related adverse events and outcome in patients treated with PD-1 inhibitors compared to those treated with PD-L1 inhibitors

	PD-1 inhibitors	PD-L1 inhibitors	*p*-value
Any grade IRAE	241 (49.4)	41 (38.3)	0.058
IRAE grade ≥ 2	178 (35.7)	24 (22.4)	**0.008**
IRAE grade ≥ 3	82 (16.5)	10 (9.3)	0.063
Multiple IRAEs	74 (14.9)	14 (13.1)	0.637
Discontinuation due to IRAEs	86 (17.3)	8 (7.5)	**0.011**

*Abbreviations*: IRAE, Immune-Related Adverse Events

p-values in bold represent a statistically significant result (< 0.05)

**Fig. 1:**
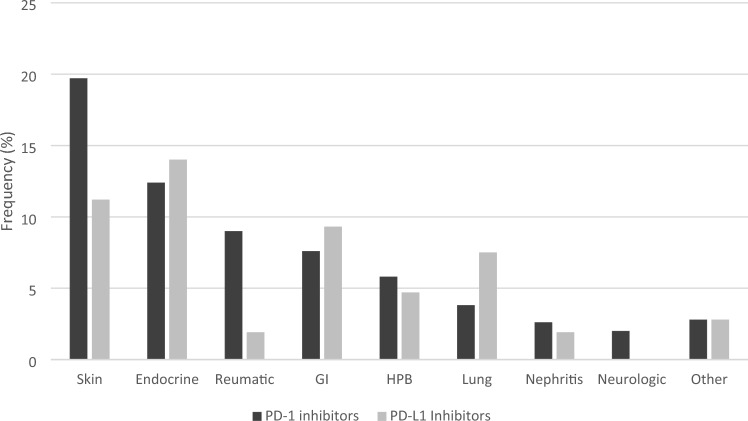
Frequency of immune-related adverse events divided by organ class/category and type of checkpoint inhibitor

Figure 1 describes the frequency of IRAEs distributed in different organs based on type of CPI. Immune-related adverse events involving the skin, the hepatopancreaticobiliary system, rheumatic conditions and nephritis were observed with numerically higher frequency among patients treated with PD-1 inhibitors. In contrast, endocrine, gastrointestinal (GI) and lung IRAEs were numerically more prevalent in patients treated with PD-L1 inhibitors. Neurological IRAEs were not reported at all for patients treated with PD-L1 inhibitors. When only grade ≥ 2 IRAEs were considered, no statistically significant differences in IRAE distribution in different organs between patients treated with PD-1 and PD-L1 inhibitors were observed.

Adjusted models on the impact of CPI type on IRAEs are demonstrated in Supplementary Table 1. Compared to PD-1 inhibitors, treatment with PD-L1 inhibitors was associated with a lower risk for both IRAEs grade ≥ 2 (OR: 0.63; 95% CI: 0.35–0.98) and discontinuation due to IRAEs (OR: 0.38; 95% CI: 0.16–0.88). No statistically significant differences between PD-1 and PD-L1 inhibitors were observed for any IRAE, IRAE ≥ 3, and multiple IRAEs. When analyses were restricted only to patients with NSCLC (as most of the patients treated with PD-L1 inhibitors had NSCLC), similar, albeit non-statistically significant, trends in OR were observed (OR: 0.63; 95% CI: 0.33—1.19 for IRAEs grade ≥ 2; OR: 0.46; 95% CI: 0.19—1.12 for discontinuation due to IRAEs).

Any grade IRAEs were significantly more common in patients with WHO PS 1 (OR: 2.99; 95% CI: 1.77–5.04) and WHO PS 2 or 3 (OR: 1.73; 95% CI: 1.07–2.81) compared to patients with WHO PS 0. Immune-related adverse events grade ≥ 2 and multiple IRAEs were significantly more prevalent in patients with WHO PS 1 (OR: 2.13; 95% CI: 1.23–3.71 for IRAEs ≥ 2; OR: 3.29; 95% CI: 1.39–7.81 for multiple IRAEs) compared to patients with WHO PS 0. Discontinuation due to IRAEs was less common in the group with other malignancies (OR: 0.50; 95% CI: 0.25–0.99) compared to malignant melanoma. Longer duration of CPI exposure was associated with higher risk for developing any IRAE, IRAEs grade ≥ 2, and multiple IRAEs.

## Discussion

Based on real-world data from a cohort of more than 600 patients with advanced solid malignancies treated with single agent CPIs, we found a favorable toxicity profile for PD-L1 inhibitors, compared to PD-1 inhibitors. Among patients treated with PD-L1 inhibitors a significantly lower frequency of IRAEs grade ≥ 2 was observed and significantly fewer had to discontinue their treatment due to IRAEs. For any grade IRAE, IRAE grade ≥ 3 and multiple IRAEs there was a trend towards less toxicity with PD-L1 inhibitors, but the difference was not statistically significant.

The finding of a favorable toxicity profile for PD-L1 inhibitors in our study cohort is consistent with the results from some [10–13] but not all [14–16] pooled analyses of randomized studies. In one meta-analysis including more than 6000 patients with NSCLC, an increased rate of any grade and high-grade IRAEs among patients receiving PD-1 inhibitors compared to those receiving PD-L1 inhibitors was found [12]. Besides, a meta-analysis of 8730 patients with various tumor types reported a similar trend, with a reduced risk of any-grade and grade ≥ 3 IRAEs associated with PD-L1 inhibitors compared to PD-1 inhibitors [11]. In our study, we observed a tendency towards fewer occurrences of any-grade IRAEs and IRAEs grade ≥ 3 with PD-L1 inhibitors. The lack of statistical significance in our results, unlike the findings in the mentioned studies, may be attributed to the substantially larger sample size in meta-analyses. On the other hand, in a pooled analysis of 3284 patients treated with CPI within clinical trials by *Pillai *et al*.,* it was noted that the incidence of overall adverse events (including non-immune related adverse events) was comparable across PD-1 and PD-L1 inhibitors. Nevertheless, they found a slightly higher occurrence of IRAEs and a doubled rate of pneumonitis from PD-1-inhibitors compared to PD-L1 inhibitors [15]. Furthermore, *Duan *et al. did not observe any significant difference in the safety profile between PD-1 and PD-L1 inhibitors in their meta-analysis of randomized clinical trials [14]. An important aspect to consider when interpreting our findings in the light of previous studies is that the existing meta-analyses, comparing toxicity from treatment with PD-1 versus PD-L1 inhibitors, have exclusively included patients from clinical trials [10–16]. This approach restricts generalizability, since patients in trials are generally younger, have less comorbidity and better PS compared to patients treated with the same agents in the real-world setting [20–22]. To our knowledge, this is the first study utilizing real-world data to compare IRAEs between treatment with PD-1 versus PD-L1 inhibitors.

Apart from differences in toxicity profiles, treatment response has also been noted to differ among patients treated with PD-1 versus PD-L1 inhibitors, with better efficacy observed for PD-1 inhibitors [14, 23, 24], although conflicting results exist [25]. The observed difference may be a result of several underlying mechanisms, reflecting the complexity of immune checkpoint blockade. Both types of antibodies block the PD-1 and PD-L1 interaction but PD-L1 inhibitors also block the binding of PD-L1 to B7-1/CD80, whereas PD-1 inhibitors also block the binding of PD-1 to PD-L2 [1, 2]. PD-L1 is widely expressed on various hematopoietic, epithelial, stromal and endothelial cells whereas PD-L2 is mainly found on hematopoietic cells as well as on certain epithelial cells, especially in the lung [2]. On the other hand, CD80 is a costimulatory molecule, with the potential of immunostimulatory effects when interacting with CD28 to activate T cells, but it also has inhibitory effects when engaging with CTLA-4, another immune checkpoint molecule. [26]. These different mechanisms of action may explain differences in effect as well as toxicity. As an additional plausible explanation for the observed clinical differences, anti-PD-1 and anti-PD-L1 antibodies differ in their affinity which may affect the depth and duration of checkpoint inhibition [26].

The WHO PS scale is widely used to evaluate the ability to tolerate chemotherapy and patients with PS ≥ 2 are considered to have low tolerance to chemotherapy [27]. Opposingly, treatment with CPIs appears safe and well tolerated for patients with PS 2 [28, 29]. This is in line with the results from our cohort where we did not observe significantly increased frequencies of IRAEs grade ≥ 2, IRAEs grade ≥ 3, or discontinuation due to IRAEs for patients with PS 2 or 3. Despite these observations, we found an increased risk of any IRAEs in patients with PS 2, which we conclude is attributed to adverse events without clinical significance, i.e. grade 1.

It has been assumed that IRAEs may differ among various malignancies, and some earlier studies have, indeed, shown a difference in IRAEs correlating to cancer type [16, 30, 31]. In the present study, however, we found no difference in the frequency of IRAEs based on cancer type, which is consistent with findings by *Wang *et al. [10]. Nevertheless, we noticed a reduced risk for treatment discontinuation due to IRAEs for the non-melanoma, non-NSCLC group compared to malignant melanoma. A plausible reason for this observation could be that patients within the non-melanoma, non-NSCLC group to a larger extent were treated with PD-L1 inhibitors.

This study has several limitations that should be considered when interpreting the results. First, the retrospective nature makes the study prone to bias, particularly misclassification bias concerning IRAE grading and information bias, due to the risk of underreporting low grade IRAEs in EMRs. To mitigate the risk for misclassification bias we applied common criteria among extractors to classify grading, but we did not provide training to extractors prior to data collection or conduct any cross-checks between extractors to ensure consistency in grading. Another limitation that could potentially impact study results is the substantial heterogeneity of cancer types between patients treated with PD-1 versus PD-L1 inhibitors, as patients treated with PD-L1 inhibitors did mainly have NSCLC, whereas patients treated with PD-1 inhibitors had a greater variance of malignancies. However, when type of malignancy was included in multivariable analysis, to mitigate this source of bias, we found no statistically significant differences between cancer types for the main study outcomes. Finally, certain variables that could potentially impact the development of IRAEs, such as ethnicity, concurrent use of immunosuppressive therapy, and pre-existing autoimmune conditions, were unavailable and therefore not included in the analyses.

In conclusion, our study including more than 600 patients with advanced cancer treated with PD-1 or PD-L1 inhibitors in a real-world setting, supports the results from randomized trials demonstrating a favorable toxicity profile for PD-L1 inhibitors versus PD-1 inhibitors. The results add evidence for clinicians in choosing the appropriate type of CPI, in clinical situations where both PD-1 and PD-L1 inhibitors are alternative treatments. Specifically, our findings suggest that PD-L1 inhibitors have lower prevalence and severity of IRAEs compared to PD-1 inhibitors, which favours anti-PD-L1 antibodies, particularly in frail patients. Moreover, we observed distinct patterns of organ-specific IRAEs, with skin-related toxicities being more common with PD-1 inhibitors and lung-related toxicities more frequently associated with PD-L1 inhibitors. This knowledge can inform clinical decisions, especially in patients with pre-existing skin or lung conditions that may increase their susceptibility to toxicities in theses organs. However, further studies utilizing large datasets from real-world data are needed to validate our findings.

## Supplementary Information

Below is the link to the electronic supplementary material.

Supplementary file1 (DOCX 15 KB)

## Data Availability

Data are available from the corresponding author upon request.

## Abbreviations

GI: Gastrointestinal
HPB: Hepato-pancreatic-biliary
PD-1: Programmed Cell Death Protein 1
PD-L1: Programmed Death-Ligand 1
