# Supplementary material for: Differences in immune-related toxicity between PD-1 and PD-L1 inhibitors: a retrospective cohort study in patients with advanced cancer
Source: Cancer Immunol Immunother. 2024 Nov 7;74(1):14. doi: 10.1007/s00262-024-03869-1 (PMC11543953; doi:10.1007/s00262-024-03869-1)
Supplement: Supplementary file 1 — Supplementary file1 (DOCX 15 KB) [file 262_2024_3869_MOESM1_ESM.docx]

Supplementary Table 1. Adjusted models on the impact of checkpoint inhibitor type on immune-related adverse events

| Parameters | Outcomes related to IRAEs | | | | |
| --- | --- | --- | --- | --- | --- |
|  | Any grade IRAE | IRAE grade ≥ 2 | IRAE grade ≥ 3 | Multiple IRAEs | Discontinuation due to IRAEs |
| Age | 1.01 (0.99 – 1.03) | 1.00 (0.99 – 1.03) | 1.01 (0.99 – 1.03) | 1.00 (0.98 – 1.03) | **1.03 (1.00 – 1.05)** |
| Gender  Male  Female | 1  1.30 (0.91 – 1.85) | 1  1.44 (0.99 – 2.07) | 1  1.25 (0.78 – 1.99) | 1  1.00 (0.61 – 1.63) | 1  0.94 (0.58 – 1.50) |
| Performance status  0  1  2 or 3 | 1  **2.99 (1.77 – 5.04)**  **1.73 (1.07 – 2.81)** | 1  **2.13 (1.23 – 3.71)**  1.47 (0.87 – 2.47) | 1  1.76 (0.85 – 3.65)  1.43 (0.71 – 2.86) | 1  **3.29 (1.39 – 7.81)**  1.88 (0.80 – 4.43) | 1  1.69 (0.83 – 3.28)  1.01 (0.52 – 1.96) |
| Cancer type  Melanoma  NSCLC  Other | 1  0.86 (0.51 – 1.44)  1.12 (0.67 – 1.86) | 1  0.83 (0.49 – 1.40)  0.97 (0.57 – 1.63) | 1  0.93 (0.48 – 1.78)  0.97 (0.50 – 1.87) | 1  0.84 (0.39 – 1.82)  1.05 (0.52 – 2.13) | 1  0.72 (0.38 – 1.37)  **0.50 (0.25 – 0.98)** |
| Line of treatment  1^st^  2^nd^  3^rd^ or later | 1  1.09 (0.71 – 1.69)  0.82 (0.47 – 1.42) | 1  1.05 (0-67 – 1.64)  0.68 (0.38 – 1.22) | 1  0.68 (0.39 – 1.21)  0.55 (0.26 – 1.19) | 1  0.81 (0.44 – 1.50)  0.84 (0.39 – 1.82) | 1  1.34 (0.75 – 2.38)  0.91 (0.40 – 2.04) |
| Type of checkpoint inhibitor  PD-1  PD-L1 | 1  0.85 (0.50 – 1.44) | 1  **0.63 (0.35 – 0.98)** | 1  0.63 (0.29 – 1.39) | 1  0.79 (0.37 – 1.69) | 1  **0.38 (0.16 – 0.88)** |
| Duration of treatment | **1.07 (1.04 – 1.10)** | **1.05 (1.02 – 1.07)** | 1.00 (0.97 – 1.03) | **1.06 (1.03 – 1.09)** | 1.00 (0.97 – 1.03) |

*Abbreviations*: IRAE, Immune-Related Adverse Events; NSCLC, Non-Small Cell Lung Cancer; PD-1, Programmed Cell Death Protein 1; PD-L1, Programmed Death-Ligand 1
